# Schuylkill Valley Veterinary Medical Association

**Published:** 1898-02

**Authors:** J. W. Sallade

**Affiliations:** Cor. Secretary


Secretary.

### SCHUYLKILL VALLEY VETERINARY MEDICAL ASSOCIATION.

THE Association held its regular quarterly meeting at the Hotel Woll, Pottsville, December 15, 1897, President Dr. Otto Noack in the chair. There were present Drs. Noack, Sallade, McCarthy, Yingst, Bieber, and Longacre. Minutes of previous meeting were read and approved. Letter of regret of his inability to be present from State Veterinarian, Dr. Leonard Pearson, was read, also rules of State Sanitary Board, and copies of act of Legislature regulating the bringing into this State of cattle for breeding purposes, sent by him, were distributed, and received the approval of the society.

A committee, consisting of Drs. Noack, Sallade, and Longacre, was appointed to draw a set of resolutions favoring meat- and milk-inspection by municipalities, to receive the indorsement of State Secretary Lee, and to be presented to city and town councils throughout the State.

Papers were read by Dr. Noack on "Milk- and Meat-Inspection as Executed by Municipalities." One by Dr. S. G. Burkholder, U. S. Service at Chicago, and a member of this society, on the subject of "Meat-Inspection as Executed by the United States," and one by Dr. Sallade on "Sanitary Science."

At the next meeting, to be held at Shenandoah, Pa., Dr. Sallade will continue his subject. Dr. McCarthy writes on "Lymphangitis," and Dr. Longacre will also produce a paper on some routine subject not yet selected.

DR. J. W. SALLADE,
Cor. Secretary.

